# Supplementary figures and images for: Myocardial architecture and patient variability in clinical patterns of atrial fibrillation
Source: Phys Rev E. Author manuscript; Available in PMC 2016 Oct 18. (PMC5068559; doi:10.1103/PhysRevE.94.042401)

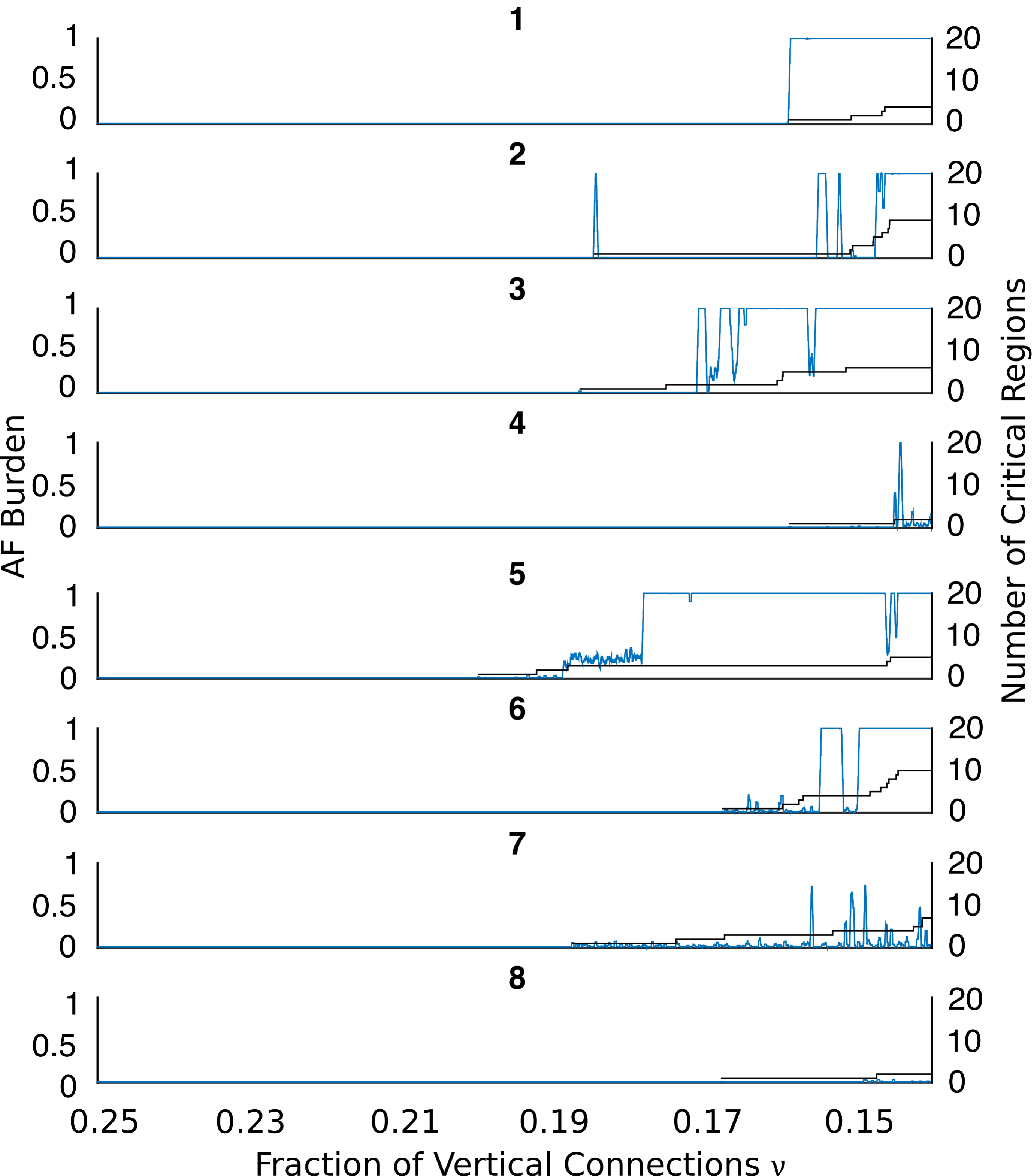

Supplement: Appendix Figure 1 [file NIHMS70146-supplement-Appendix_Figure_1.pdf]

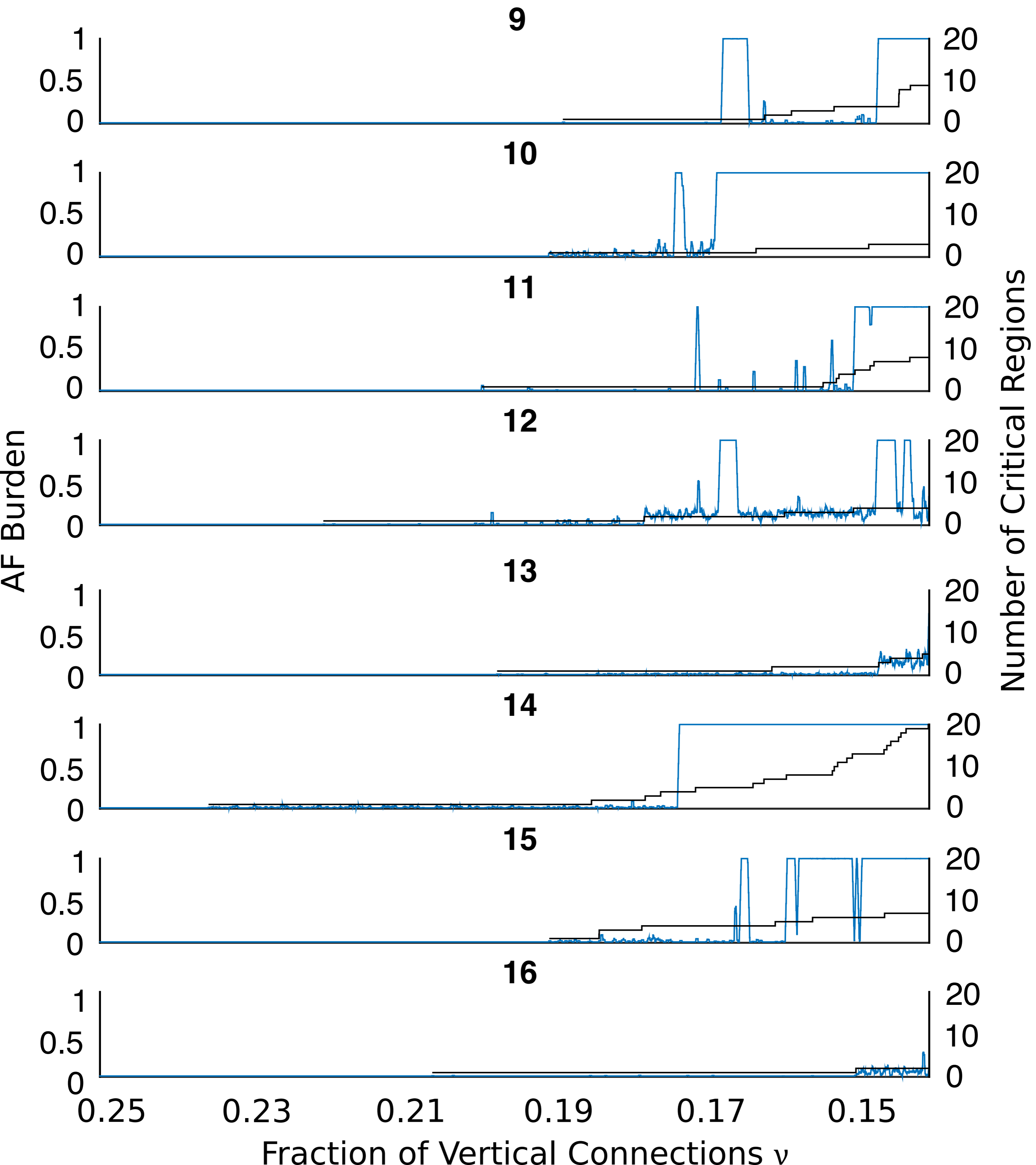

Supplement: Appendix Figure 2 [file NIHMS70146-supplement-Appendix_Figure_2.pdf]

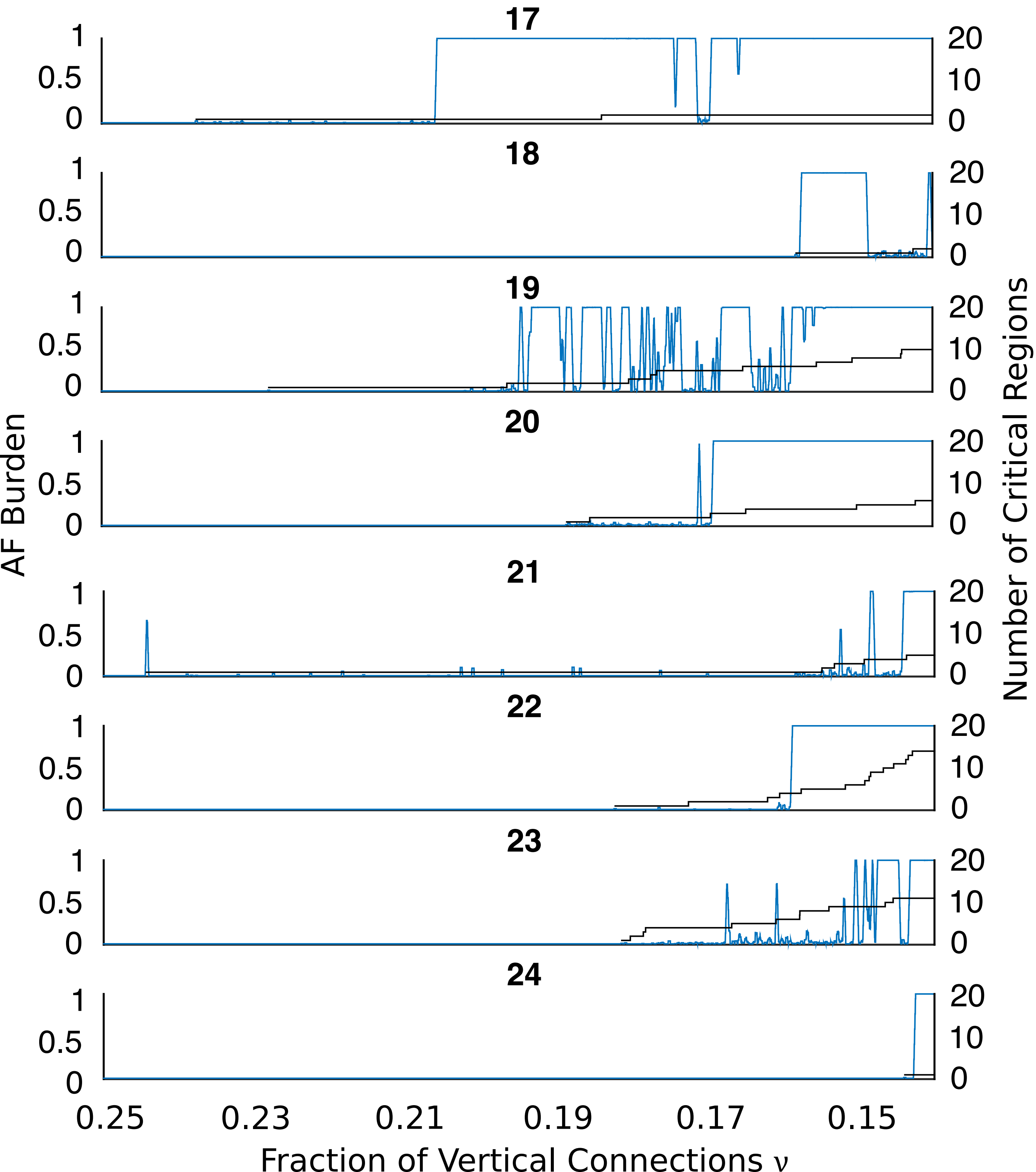

Supplement: Appendix Figure 3 [file NIHMS70146-supplement-Appendix_Figure_3.pdf]

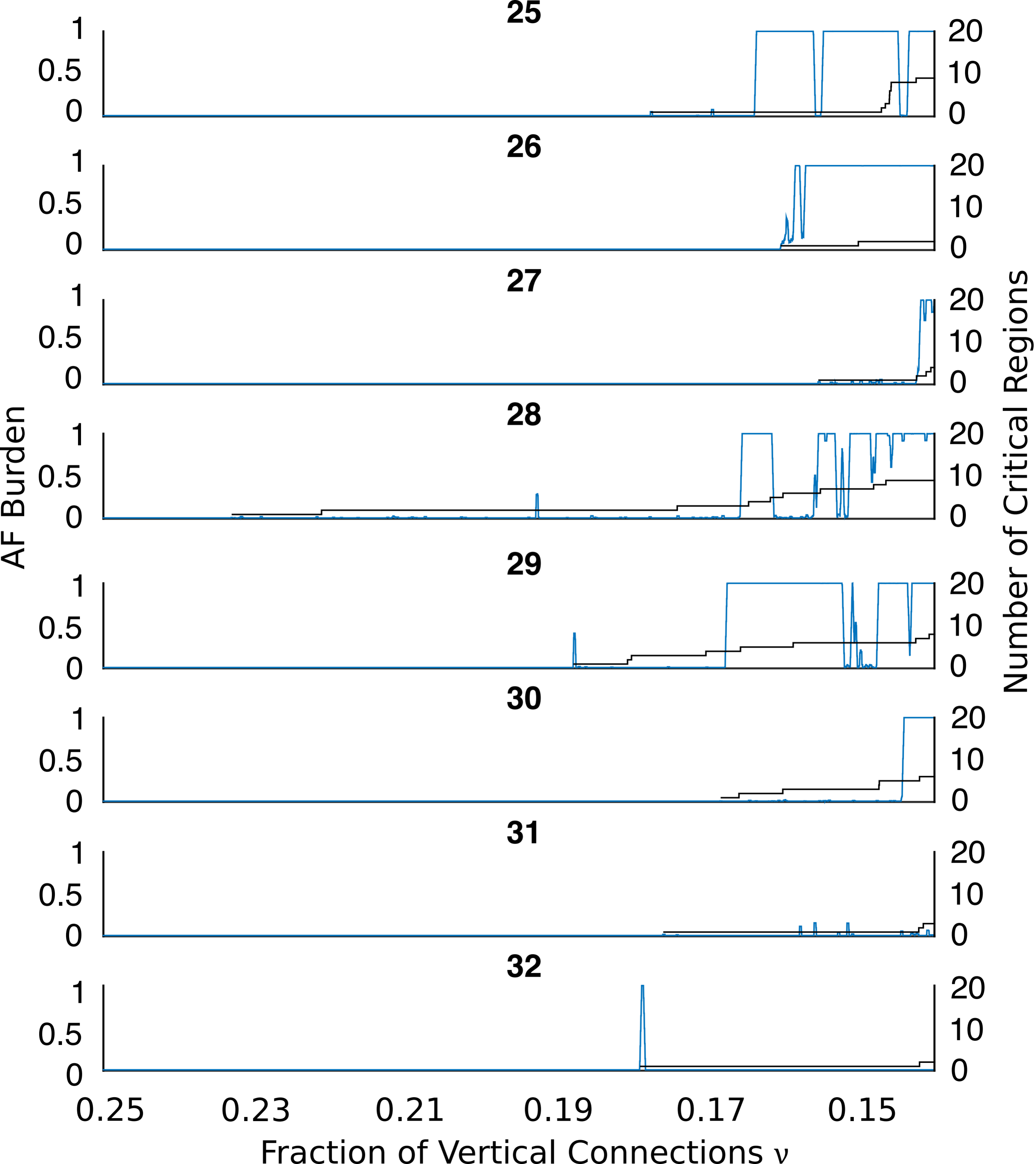

Supplement: Appendix Figure 4 [file NIHMS70146-supplement-Appendix_Figure_4.pdf]

# Simulations 1 to 8

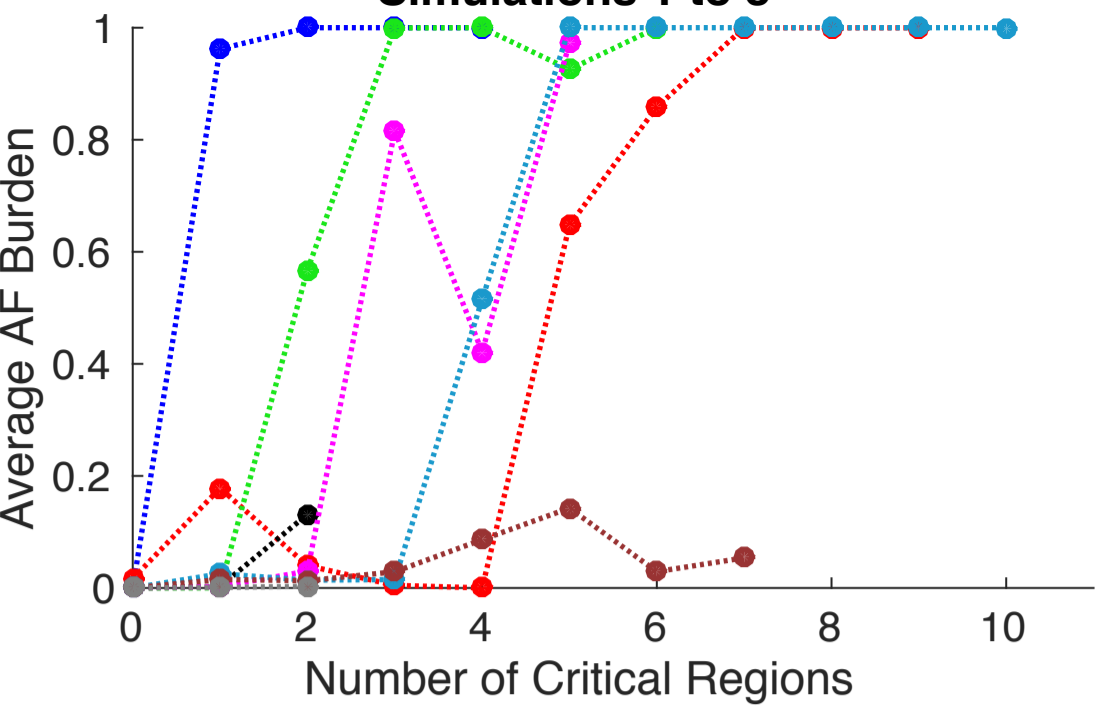

## Simulations 9 to 16

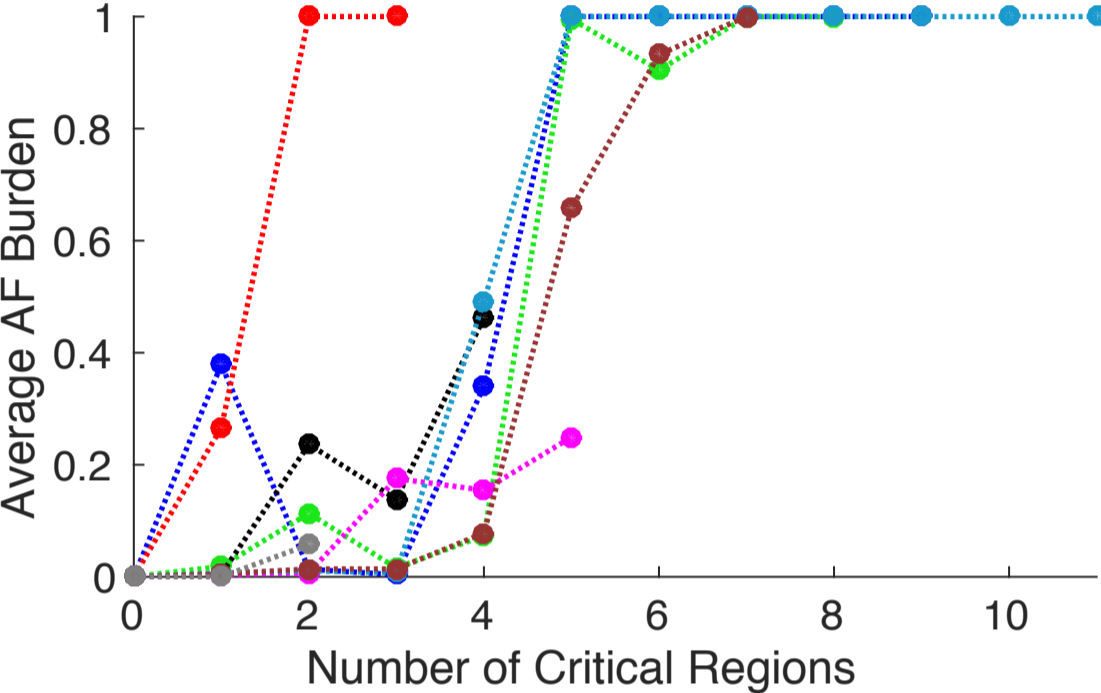

## Simulations 17 to 24

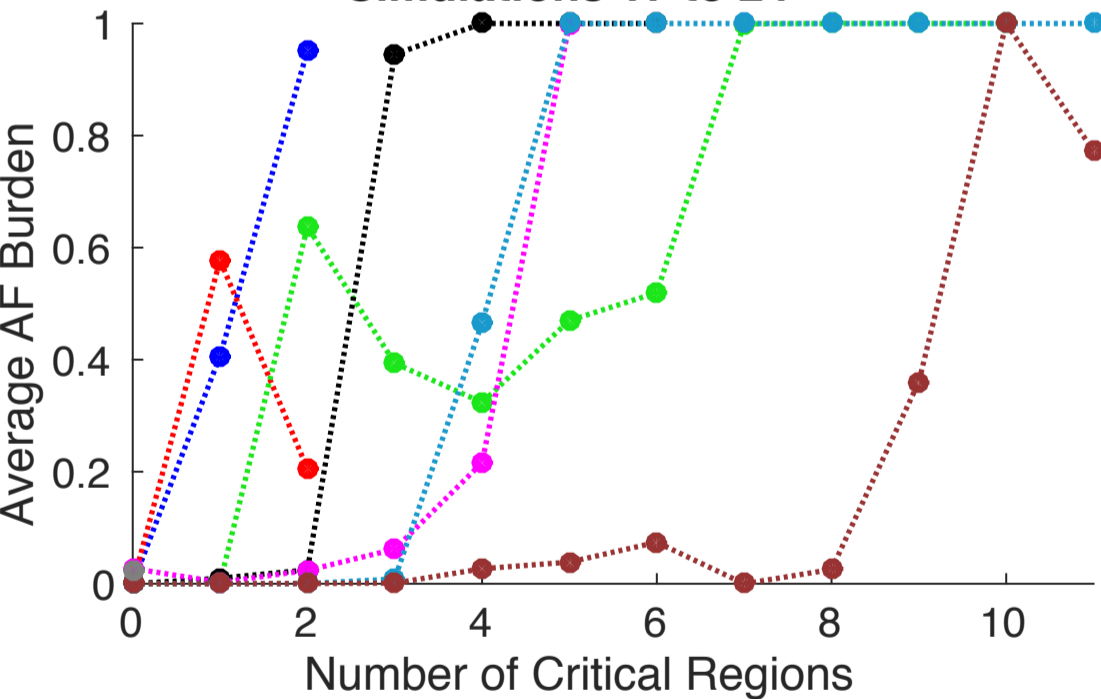

## Simulations 25 to 32

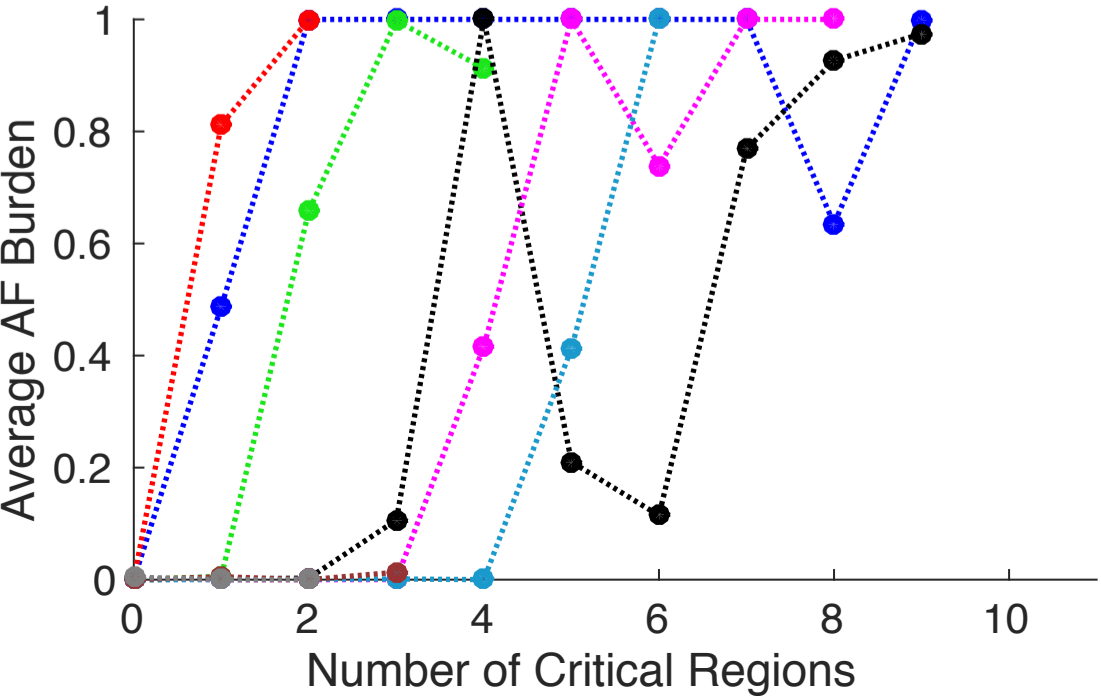

Supplement: Appendix Figure 5 [file NIHMS70146-supplement-Appendix_Figure_5.pdf]
