# Supplementary material for: Myocardial architecture and patient variability in clinical patterns of atrial fibrillation
Source: Phys Rev E. Author manuscript; Available in PMC 2016 Oct 18. (PMC5068559; doi:10.1103/PhysRevE.94.042401)
Supplement: Appendixes [file NIHMS70146-supplement-Appendixes.pdf]

APPENDIX A: FULL DESCRIPTION OF THE MODEL

We provide a brief summary of the model and parameters used. The tissue is represented by an $L \times L$ square grid of discrete cells. Heart muscle tissue forms a branching network of interacting cells where each cell is coupled to its neighbors longitudinally and to its neighbors transversally with probability ν. The structure of the lattice can be considered to be a set of aligned longitudinal cables of single cell thickness that are coupled to neighboring cables with frequency ν. The lateral uncoupling of cells is thus represented by reducing the frequency of vertical couplings through the parameter ν. To represent the simplest features of an atrium we apply periodic boundary conditions vertically and open boundary conditions horizontally to the two-dimensional model, giving a cylindrical topology, which is a convenient substrate to study the evolution of wave fronts. Cells along the left boundary are pacemakers and self-excite at a fixed period $T' = 660$ ms to mimic normal cardiac rhythm.

Simplifying the action potential, a cell may be in one of three states: resting (repolarized), excited (depolarizing), or refractory. An excited cell causes neighboring coupled resting cells to become excited. Thus the wave front is a coherent propagation of this excitation throughout the tissue. A healthy atrial area is $L' \times L' \approx 20$ cm^2. Atrial muscle tissue is made of nearly cylindrical myocytes of length $\Delta x' \approx 100\ \mu$m and diameter $\Delta y' \approx 20\ \mu$m packed in an irregular brick-wall-like pattern. The time taken for a cell to depolarize, $\Delta t' \approx 0.6$ ms, is much shorter than the refractory period $\tau' \approx 150$ ms.

Cellular electrical dysfunction of any cause can introduce a degree of noise into the excitation process. A fraction of randomly chosen cells in the lattice is chosen to be dysfunctional. Such cells have a finite probability of not exciting in response to an excited neighbor and hence can block the propagation of activation.

Translating real tissue values into the model gives $L = L'/\Delta x' = 1000$. We coarse grain the model by taking $\Delta x' \to b\Delta x'$, where b is the number of cells within a unit of space in the model. As the conduction velocity $\theta_x' = \Delta x'/\Delta t' \approx 0.2$ ms^{-1} is fixed, we also find $\Delta t' \to b\Delta t'$. We take $b = 5$, implying that the parameters of the model are system size $L = 200$, refractory period $\tau = \tau'/b\Delta t' = 50$, the fraction of dysfunctional cells $\delta = 0.05$, the probability of dysfunction $\epsilon = 0.05$, and the period of pacing $T = T'/b\Delta t' = 220$ using $T' = 660$ ms, leaving the fraction of transverse connections ν as the only control parameter. See our previous paper [12] for a detailed discussion on the model.

APPENDIX B: 32 SIMULATED PATIENTS

We present the results of all 32 simulated patients. We start at $\nu = 0.25$, where all the simulated heart muscle tissue is in SR, but as we lower ν, fibrillation emerges. The initial conditions for each patient are created by assigning the same number of vertical connections (identical initial ν) but at different random positions. Next the accumulation of cellular uncoupling is implemented by reducing ν (e.g., to mimic the progression of fibrosis or gap junctional uncoupling). To do this, we run simulations for a period of $T = 4.3 \times 10^7$ time steps in the computer model and vertical connections are removed at a rate of one connection every 9000 time steps.

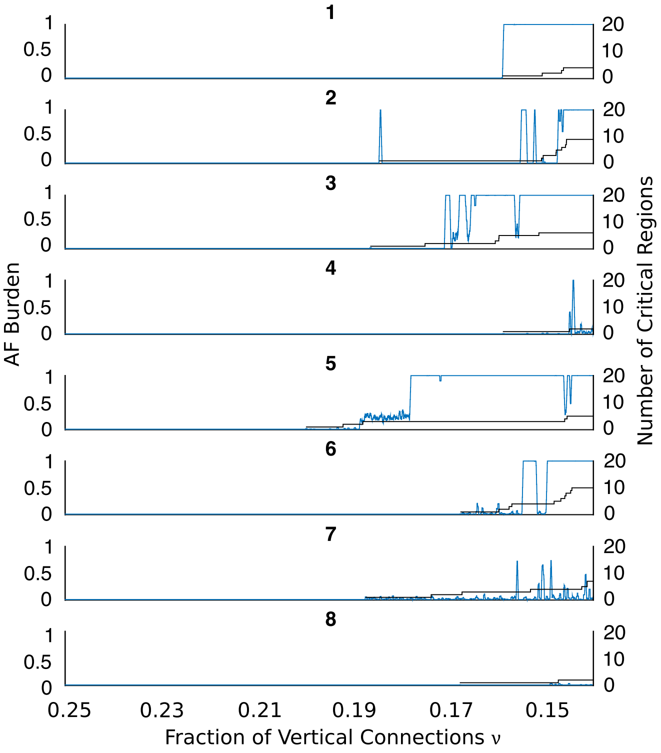

FIG. 5. The AF burden (blue) varies with time, calculated from the time in AF in a sliding window of 5×10^6 time steps for simulations 1–8. Also shown is the accumulation of initiating critical regions (black curve) versus the fraction of vertical connections.

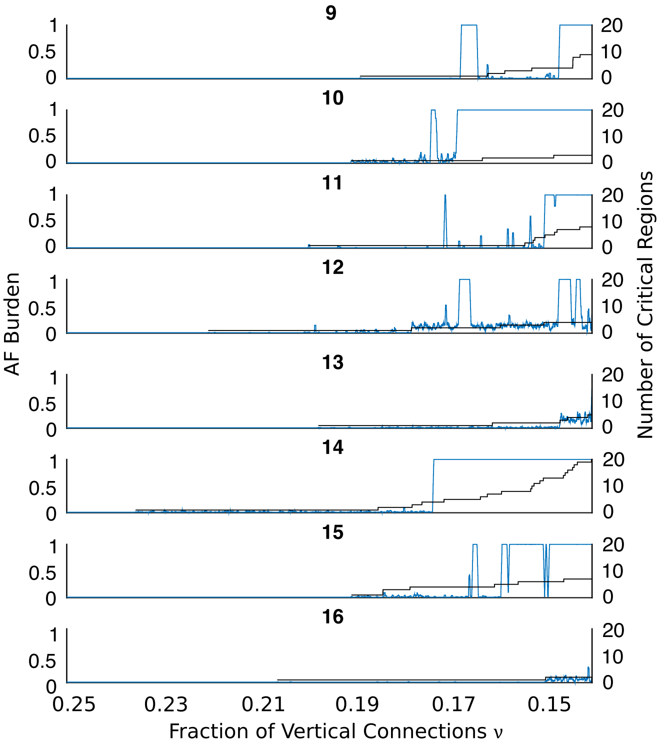

FIG. 6. The AF burden (blue) varies with time, calculated from the time in AF in a sliding window of 5×10^6 time steps for simulations 9–16. Also shown is the accumulation of initiating critical regions (black curve) versus the fraction of vertical connections.

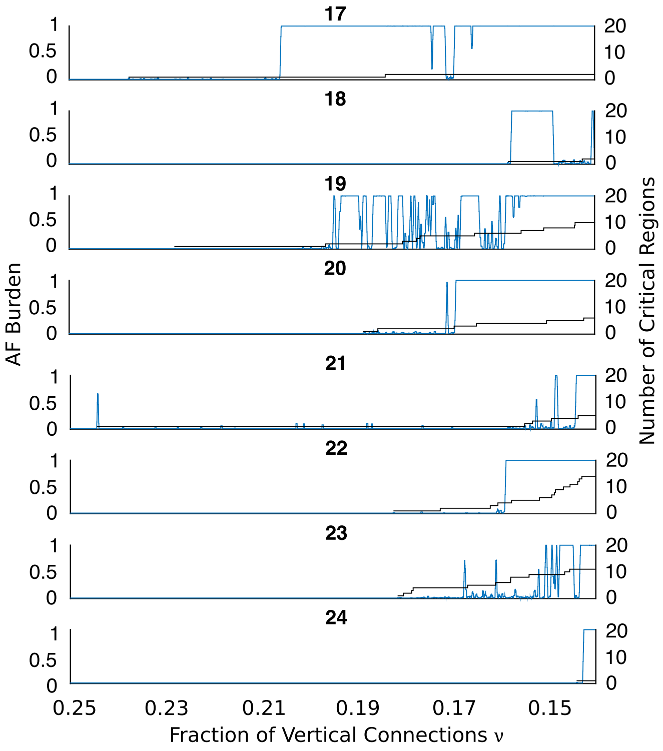

FIG. 7. The AF burden (blue) varies with time, calculated from the time in AF in a sliding window of 5×10^6 time steps for simulations 17–24. Also shown is the accumulation of initiating critical regions (black curve) versus the fraction of vertical connections.

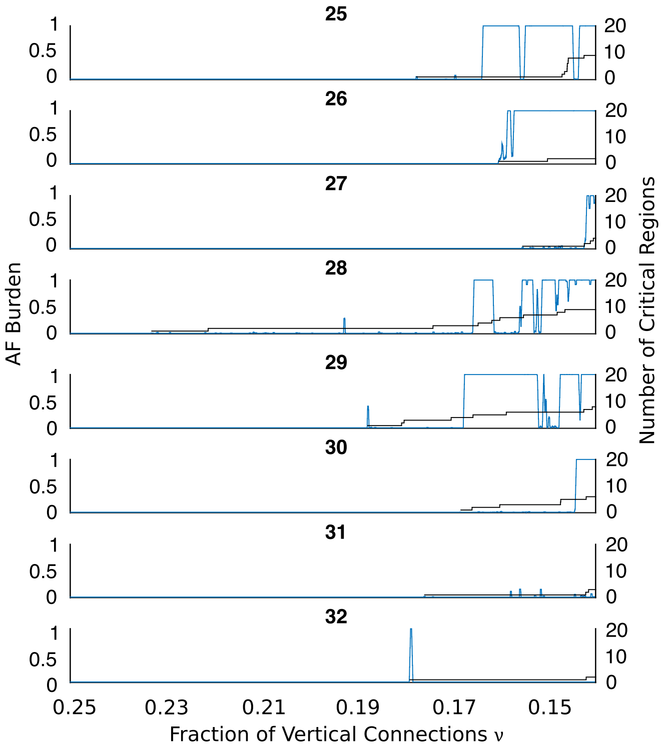

FIG. 8. The AF burden (blue) varies with time, calculated from the time in AF in a sliding window of 5×10^6 time steps for simulations 25–32. Also shown is the accumulation of initiating critical regions (black curve) versus the fraction of vertical connections.

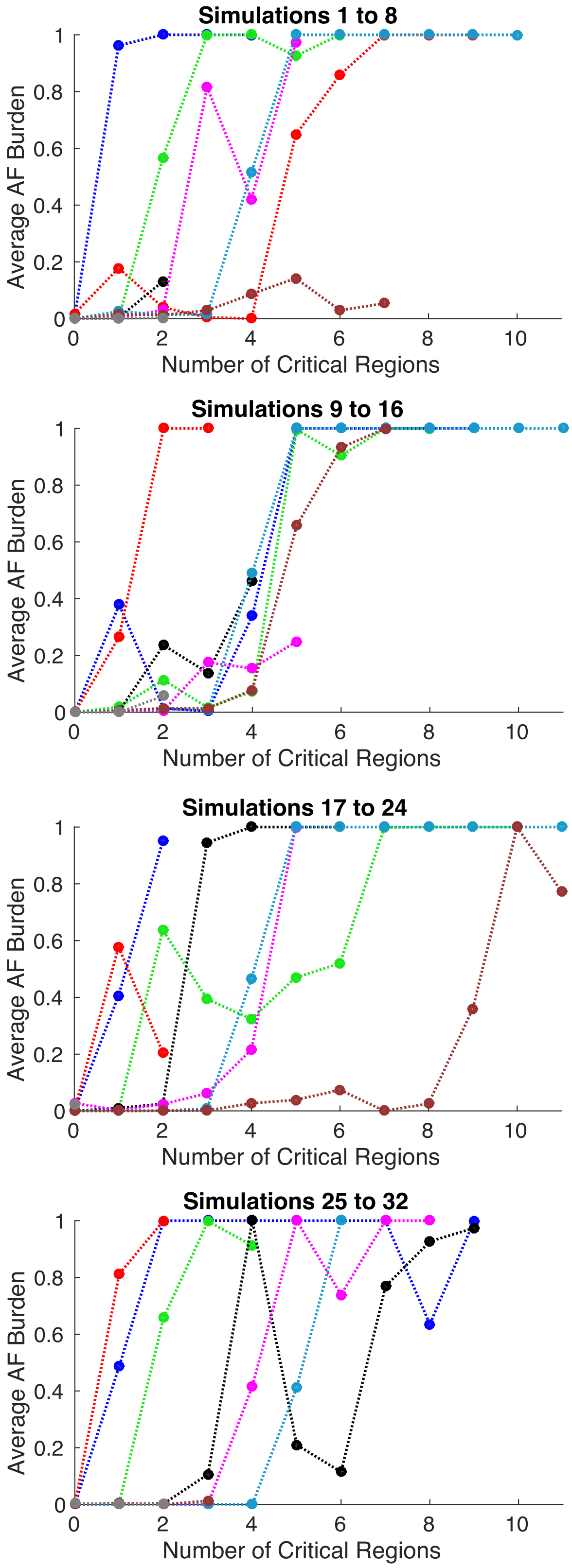

FIG. 9. Average AF burden for all 32 simulations as a function of the number of critical regions. The standard deviations and standard error are too small to be seen at this scale. The differences in the accumulation of critical regions between patients better predict the variability in the AF behavior observed.

We observe the dynamics of excitation wave fronts as the transversal uncoupling accumulates in the tissue. All other model parameters are set to physiological values: $L = 200$, $\tau = 50$, $\epsilon = 0.05$, and $\delta = 0.05$. Pacemaker cells self-activate with a period of $T = 220$.

The AF burden and number of critical regions versus fraction of vertical connections are shown in Figs. 1–4. We show the average AF burden as a function of the number of critical regions for each simulation in Figs. 5–9.
